# Smoking data quality of primary care practices in comparison with smoking data from the New Zealand Māori and Pacific abdominal aortic aneurysm screening programme: an observational study

**DOI:** 10.1186/s12889-024-19021-8

**Published:** 2024-06-05

**Authors:** Karen Bartholomew, Phyu Sin Aye, Charlotte Aitken, Erin Chambers, Cleo Neville, Anna Maxwell, Peter Sandiford, Aivi Puloka, Sue Crengle, Katrina Poppe, Robert N Doughty, Andrew Hill

**Affiliations:** 1 Service Improvement and Innovation, Health New Zealand Te Whatu Ora, Auckland, New Zealand; 2 University of Auckland, Auckland, New Zealand; 3 University of Otago, Dunedin, New Zealand

**Keywords:** Smoking, Tobacco, Data quality, Risk factor, Screening

## Abstract

**Background:**

Quality smoking data is crucial for assessing smoking-related health risk and eligibility for interventions related to that risk. Smoking information collected in primary care practices (PCPs) is a major data source; however, little is known about the PCP smoking data quality. This project compared PCP smoking data to that collected in the Māori and Pacific Abdominal Aortic Aneurysm (AAA) screening programme.

**Methods:**

A two stage review was conducted. In Stage 1, data quality was assessed by comparing the PCP smoking data recorded close to AAA screening episodes with the data collected from participants at the AAA screening session. Inter-rater reliability was analysed using Cohen’s kappa scores. In Stage 2, an audit of longitudinal smoking status was conducted, of a subset of participants potentially misclassified in Stage 1. Data were compared in three groups: current smoker (smoke at least monthly), ex-smoker (stopped > 1 month ago) and never smoker (smoked < 100 cigarettes in lifetime).

**Results:**

Of the 1841 people who underwent AAA screening, 1716 (93%) had PCP smoking information. Stage 1 PCP smoking data showed 82% concordance with the AAA data (adjusted kappa 0.76). Fewer current or ex-smokers were recorded in PCP data. In the Stage 2 analysis of discordant and missing data (*N* = 313), 212 were enrolled in the 29 participating PCPs, and of these 13% were deceased and 41% had changed PCP. Of the 93 participants still enrolled in the participating PCPs, smoking status had been updated for 43%. Data on quantity, duration, or quit date of smoking were largely missing in PCP records. The AAA data of ex-smokers who were classified as never smokers in the Stage 2 PCP data (*N* = 27) showed a median smoking cessation duration of 32 years (range 0–50 years), with 85% (*N* = 23) having quit more than 15 years ago.

**Conclusions:**

PCP smoking data quality compared with the AAA data is consistent with international findings. PCP data captured fewer current and ex-smokers, suggesting ongoing improvement is important. Intervention programmes based on smoking status should consider complementary mechanisms to ensure eligible individuals are not missed from programme invitation.

**Supplementary Information:**

The online version contains supplementary material available at 10.1186/s12889-024-19021-8.

## Background

Tobacco smoking is a known major risk factor for many common diseases, such as lung cancer, chronic obstructive pulmonary disease (COPD), and cardiovascular disease (CVD) [1]. Smoking accounted for 9.59% of all illness, disability and premature mortality in New Zealand in 2019 [2, 3]. Prevalence of current smokers has declined from 18% in 2011-12 to 8.3% (i.e. 350,000 adults approximately) in 2022-23; however, the risk of developing smoking-related diseases remains as about one million New Zealanders are ex-smokers [4]. New Zealand shows profound ethnic differences in smoking prevalence and impact of smoking. Māori (Indigenous) and Pacific Peoples are more likely to smoke (3.3 and 1.3 times more likely respectively), compared with non-Māori, non-Pacific Peoples [4]. In terms of smoking-related disease, Māori were 3.2 times more likely to die from lung cancer, and 1.9 times more likely to die from ischemic heart disease in 2019, than the non-Māori population [5].

Smoking history is an important criterion in risk assessment and screening for smoking-related diseases as well as critical information for smoking cessation initiatives. Smoking status (categorised as current smoker, ex-smoker or never smoker) is included in the PREDICT-1 equations for the Cardiovascular Disease Risk Assessment (CVDRA) [6], which is a key enabler activity in primary care for improved cardiovascular risk management [7]. Smoking information, such as years smoked, number of cigarettes per day and time since smoking cessation, is also used as criteria for eligibility or as a core component of risk assessment (e.g., the PLCOm2012 model) for lung cancer screening internationally [8]. In New Zealand, members of our health equity research group are currently trialling lung cancer screening by using PLCOm2012 [9]. Smoking is also a key risk factor for Abdominal Aortic Aneurysm (AAA) [10]. Members of our research group are also leading a broad research programme testing specific policy-relevant information for consideration of Māori and Pacific AAA screening programme in New Zealand. This AAA programme is currently considering the potential for targeting national screening by using smoking as an eligibility criterion in the future [11]. Additionally, smoking cessation programmes rely on smoking status records to offer smoking cessation supports to smokers. The New Zealand Ministry of Health has incentivised primary care targets, updated in 2015 and ongoing; one of which is 90% of smokers being offered brief advice to quit including access to cessation services [12].

Reliable information on individuals’ smoking history is essential for smoking-related interventions to effectively reach those most at risk. Smoking information is collected as part of usual health care in primary care practices (PCP). As of July 2022, 94% of New Zealanders were enrolled in a PCP [13]; 83% of 55–64 year-olds and 87% of 65–74 year-olds reported having contact with their general practitioner (GP) in the last 12 months in 2021/22, so did 70.5% of Māori, 66.6% of Pacific, 67% of Asian, and 78.5% of European/Other populations [14]. New Zealand has set a target of recording smoking information in 90% of enrolled people [15]. Previous audits of PCP electronic medical records showed that the completeness of smoking information has improved from being recorded in less than 50% of patients up to 95% of patients over the past two decades [16–20]. There are known issues with smoking data completeness (missing data) in electronic medical records, as well as potential issues with smoking data accuracy [21]. Issues with accuracy may arise due to changes in patient smoking status over time, patient under-reporting (smoking stigma or recall bias), and practitioner or IT differences in data capture and recording. Smoking data accuracy related to disease risk is also affected by lack of collection for example of second-hand exposure, duration of smoking, inter-individual variation in smoking technique and dose differences by product type [22–24].

Little is known about the accuracy of smoking data in New Zealand PCPs despite the importance of smoking status to individual health risk, and eligibility for potential interventions relevant to that risk. This paper seeks to provide insight into primary care smoking data quality through comparison of smoking status recorded in PCPs to that in the AAA screening programme [11]. The aim of the study is to quantify the concordance of PCP smoking status against the AAA reference at baseline, and to quantify any change in current smoking status for those identified with discordant or missing data, who may not be identified as eligible for risk-based interventions.

## Methods

### Study design

This project retrospectively reviewed the smoking data recorded in primary care practice (PCP) patient management systems (PMS) and compared it to the smoking data prospectively collected in the Māori and Pacific AAA screening research programme.

It involved two stages: Stage 1 included comparison of PCP smoking status recorded close to the time of AAA screening with AAA smoking status collected from participants at the screening session. Stage 2 involved review of PCP longitudinal smoking data for people who were potentially misclassified as never or ex-smoker or had missing smoking status in the Stage 1 of the project, comparing their latest PCP smoking status with AAA smoking status. This project was undertaken as a data quality improvement project to consider the merit of updating PCP smoking status with the AAA programme data.

This project has been reported according to the strengthening the reporting of observational studies in epidemiology (STROBE) statement (supplementary Table 3) [25].

### Data sources

The data for this project was obtained from the Māori and Pacific AAA screening research programme electronic database and the PCP PMS for AAA screened participants. Accurate linkage between data sources was achieved through individual participants’ national health index identifier, a unique and unchanging alphanumeric string assigned to each person when they first access the healthcare system in New Zealand.

The Māori and Pacific AAA screening programme, started in 2016, had screened for AAA for approximately 2500 Māori and 680 Pacific Peoples who live in the main urban Auckland and Waitematā regions. Eligible people from 21 Auckland and 19 Waitematā PCPs were invited to participate. Over the course of the project, AAA participants were recruited from around 15% of Auckland and 20% of Waitematā PCPs. AAA screening is a one-off limited-view abdominal ultrasound that records transverse and longitudinal aortic diameters and refers participants with aneurysms into local vascular services to assess for repair or surveillance. There are known inequities in AAA with a higher mortality for both Māori and Pacific Peoples in New Zealand [26–29]. The screening cohort initially included Māori participants – men aged 60–74 and women 65–74 (55–74 for men and 60–74 for women at the initial pilot of 500). It expanded to include Pacific men in 2020. Inclusion criteria are based on age and ethnicity. Exclusions include previously diagnosed AAA, or health conditions that may limit the benefits from early detection of an AAA. Recruitment into the AAA screening programme is independent of smoking status.

For Stage 1 of the project, with agreement of the Primary Health Organisation (PHO) and the participating PCP, smoking status was extracted retrospectively from the PMS of PCPs who used Medtech (MedTech 32 or MedTech Evolution) by a third-party specialist primary care vendor using specified READ Codes (Supplementary Table 1). Data were extracted manually from PCPs that used other PMS’s (MyPractice or Incidi). These PMS’s represent those used across PCPs in Auckland, with Medtech being the most common PMS in use at the time. Extraction of smoking data occurred as part of usual AAA programme procedures where smoking data and other relevant clinical data were extracted.

For Stage 2, the PCP longitudinal smoking data was collected from PCPs who agreed to participate and was sourced manually as a case review from various locations of individual patient records in the PMS, e.g., diagnosis, screening, classifications, history, and patient dashboard, where smoking status would have been recorded using codes.

AAA screening smoking status was recorded by the Kaimatawai Puku (AAA screeners) during the screening session as part of the health questionnaire prior to their abdominal ultrasound examination. As per the New Zealand Ministry of Health definitions [4, 30, 31] the smoking status was classified as people who have smoked (defined as smoked > 100 cigarettes in lifetime) including daily smoker (smoke every day), current smoker and occasional smoker (smoke at least monthly), ex-smoker (stopped > 1 month ago) or never smoker (< 100 cigarettes in lifetime). Information was also collected on smoking start age, average tobacco use per day, smoking duration, duration of smoking cessation (if ex-smoker) and information on passive smoking using a standardised set of questions.

Age, sex and ethnicity data were obtained from the PCP records. Age at screening was calculated from date of birth, categorised into 10-year groups: <60, 60–69, and 70–79 years. Sex was categorised into male and female. Recording of ethnicity data followed the Ethnicity Data Protocols for Health and Disability Sector of Aotearoa New Zealand [32]. Mortality data had been updated linking to the national Mortality Collections [33].

### Study procedure

In Stage 1, data included were the smoking status extracted from PCPs prior to their screening session for people who were screened in the AAA programme. This occurred in the period from May 2016 to February 2018, and July-August 2020. AAA smoking status was extracted from the AAA screening data, recorded at the time of screening session. A comparative analysis was then performed to analyse concordance and reliability of the two smoking datasets.

In Stage 2, the following discordant or missing data from Stage 1 who may not be identified as eligible for risk-based interventions were identified for review of their PCP longitudinal smoking data: (1) those documented as never smokers in the PCP but as a current or ex-smoker at AAA screening; (2) those documented as ex-smokers in the PCP but as current smokers at AAA screening; and (3) those with missing PCP smoking data at the time data were extracted. The managers or directors of the PCPs where participants were enrolled were contacted via email and phone to arrange for a member of the AAA team to visit the PCPs and extract each participant’s most recent smoking data from their PMS. These visits were completed between August and October 2022. During the PCP visits, information was collected as field notes and informally during discussions with PCP staff on where smoking status was recorded within the PMS, as well as primary care staff perspectives on barriers and enablers for collecting quality smoking data. Content analysis [34] was used to categorise notes taken during the audit visits.

### Smoking data coding

To enable comparison of smoking data between the PCP and AAA datasets, the different smoking status codes were mapped to the following three groups using the same Ministry of Health definitions [31]: (1) Current smoker – daily smoker (D) or occasional smoker (O) from the AAA data; 137R current smoker, 137G trying to give up smoking, 1373 light smoker – 1–9 cigs/day, 1375 heavy smoker – 20–39 cigs/day, 1374 moderate smoker – 10–19 cigs/day from the PCP data. (2) Ex-smoker – ex-smoker from the AAA data; 137 S ex-smoker – more than 1 year, 137 K stopped smoking – less than 1 year, 137 F ex-smoker – amount unknown from the PCP data. (3) Never smoker – never smoked (< 100 cigarettes in lifetime) from the AAA data; and 1371 never smoked tobacco from the PCP data.

### Data analysis

For the data quality assessment, we examined accuracy (cross-sectional concordance) with AAA data as the reference comparator. The rationale for considering AAA smoking data as the reference comparator is that it has been collected using the standard smoking definitions, questions were in the reference format used by the Ministry of Health national surveys, multiple standard smoking related information were collected, data collectors collected information directly from the participants and were consistent over time, and data was collected into one reference database.

The data were summarised using frequencies and percentages. T test was used to analyse the age difference, and the two-tailed mid P test was used to analyse the differences in prevalence, with analyses of missing data frequency stratified by the practice participation status. Inter-rater reliability was assessed using the weighted (to account for the ordered nature of the data) Cohen’s kappa score [35]. Analyses were performed using Microsoft Excel, Open Epi and Stata v16.

### Ethics approval

The AAA screening programmes have ethical approval from the national Health and Disability Ethics Committee (HDEC, 15/NTB/47 & 19/NTB/227) and include a process of participant informed consent developed by a Māori health literacy specialist. This audit was considered low risk and did not require additional project specific HDEC approval. Local research office locality approval for the AAA programme was provided via the Research and Knowledge Centre, Te Whatu Ora, Waitematā (RM14588 and RM12997) and local research governance approvals were provided by participating Primary Health Organisations. Individual PCP agreement was also obtained. Informed consent was obtained from all participants involved in the AAA screening programme, including specific consent for access to primary care smoking records.

## Results

The results have been outlined for the two stages of the review, examining the accuracy of the recorded cross-sectional PCP smoking status against the AAA smoking status from Stage 1 and then examining the longitudinal change and currently recorded smoking status for a subgroup of Stage 1 participants.

### Stage 1: comparison of PCP smoking status and AAA smoking status

A total of 1841 people who underwent AAA screening and were from the eligible PCPs were included in the audit. A majority were 60–69 years (69%), male (73%), and Māori (63%) among their counterparts (Table 1). Of the total 1841 people, 1716 (93%) had PCP smoking data, showing a similar distribution: 69% for 60–69 years, 74% for male, and 61% for Māori.

**Table 1:** Demographics of participants who underwent AAA screening

		Stage 1 PCP Smoking Status		Total
		Recorded	Not Recorded	
Age (at screening)				
	< 60 years	118 (7%)	1 (1%)	119 (6%)
	60–69 years	1,180 (69%)	92 (74%)	1,272 (69%)
	70–79 years	418 (24%)	32 (26%)	450 (24%)
Gender				
	Male	1,266 (74%)	82 (66%)	1,348 (73%)
	Female	450 (26%)	43 (34%)	493 (27%)
Ethnicity				
	Māori	1,049 (61%)	114 (91%)	1,163 (63%)
	Pacific	667 (39%)	11 (9%)	678 (37%)
Total		1,716	125	1,841

Of these 1716 recorded, 1415 people (82%; 95%CI 80.6–84.2%) had concordant smoking status between PCP data and AAA screening data (Table 2), showing a weighted kappa score of 0.76. Disagreements (*n* = 301) between PCP and AAA screening data were contributed by the PCP data not capturing current or ex-smokers (*n* = 188; 62%) identified at the time of AAA screening. Of the 1716 people, the prevalence of current smoker was 18% (317/1716) in PCP versus 20% (349/1716) in AAA screening (*p* = 0.1676); and that of ex-smoker was 48% in PCP versus 49% in AAA screening (*p* = 0.539) (Table 2).

**Table 2:**
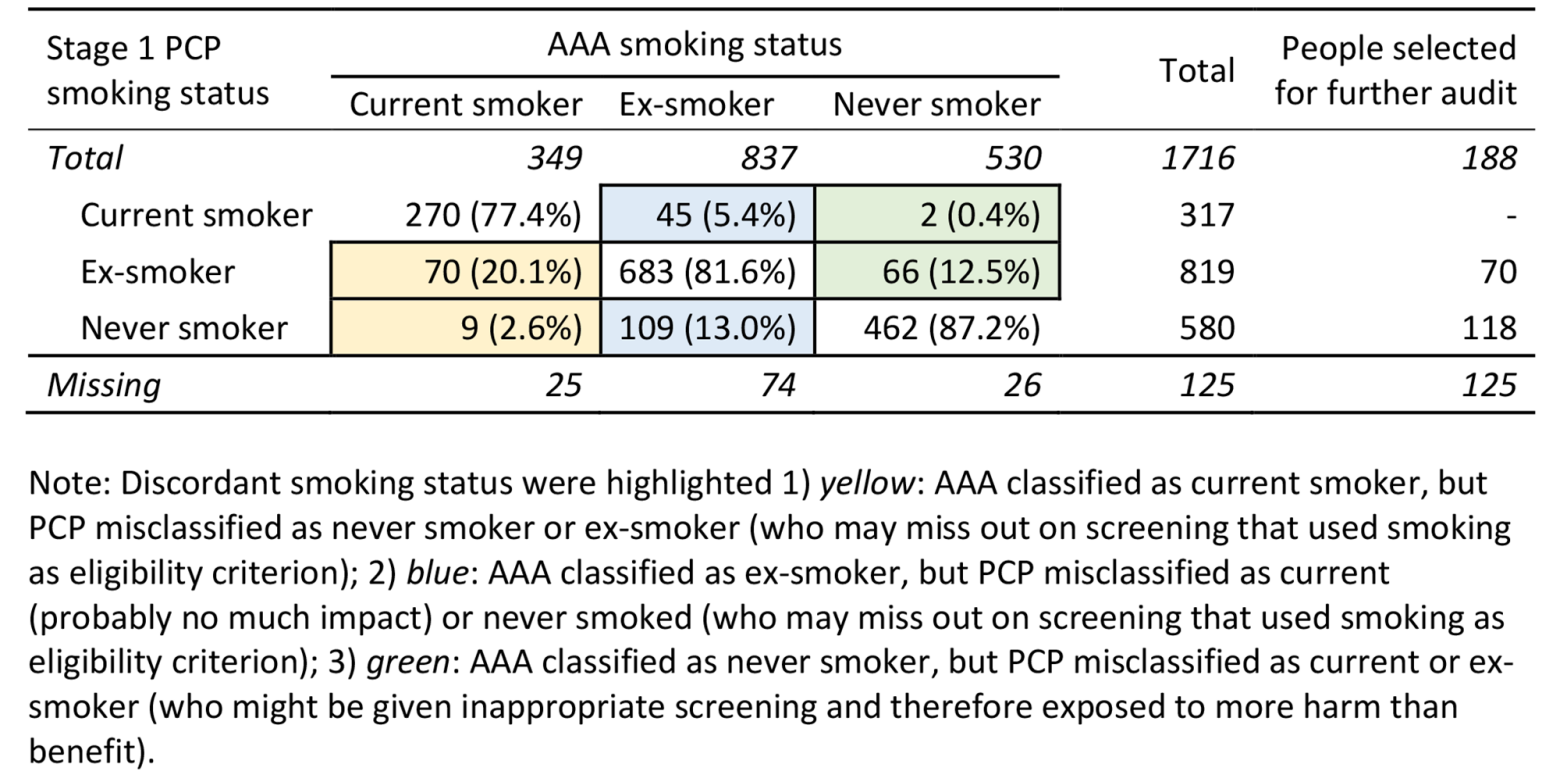
Concordance of Stage 1 PCP smoking status (recorded around the time of AAA screening) and AAA smoking status (collected at AAA screening)

Of those 349 classified as current smoker in AAA, PCP classified 79 (22.7%) as never smoker or ex-smoker (Table 2 yellow highlighting). Of those 837 ex-smokers in AAA, PCP misclassified 45 (5.4%) as current, and 109 (13%) as never smoker (Table 2 blue highlighting). Of those 530 who were never smokers in AAA, PCP classified 68 (12.9%) as current or ex-smoker (Table 2 green highlighting).

### Stage 2: PCP longitudinal smoking data

The 313 participants who were erroneous never smoker or ex-smoker (*n* = 188) or had missing smoking status (*n* = 125) in the Stage 1 PCP data were enrolled at 37 different PCPs across the study region. Of these, 29 PCPs, accounting for 212 (68%) participants, agreed to take part in Stage 2 of the project. Five PCPs did not respond (representing 84 participants) and three practices (17 participants) were unable to take part (Table 3). Individuals from participating PCPs had similar age to those from non-participating PCPs (mean 67 versus 66 years), had a more even gender mix (65% versus 93% male, *p* < 0.0001) and a much higher proportion of Māori relative to Pacific (91% versus 23%, *p* < 0.0001). Most Pacific participants (80% of *n* = 98) were enrolled in Stage 2 non-participating PCPs. As all the Pacific participants were men, they contributed to the high proportion of males (93%) in non-participating PCPs.

**Table 3:** Review of PCP longitudinal smoking data for the cohort of people with discordant smoking status who were audited

			Stage 1 PCP smoking status			
			Ex-smoker N (%)	Never smoker N (%)	Missing N (%)	Total N (%)
Total Stage 1 PCP smoking status			70	118	125	313
Total participants from the 8 practices who were unable to take part or did not respond			25	61	15	101
	Age					
		< 60 years	1 (4)	3 (4)	1 (7)	5 (5)
		60–69 years	18 (72)	42 (69)	13 (87)	73 (72)
		70–79 years	6 (24)	16 (26)	1 (7)	23 (23)
	Gender					
		Male	23 (92)	57 (93)	14 (93)	94 (93)
		Female	2 (8)	4 (7)	1 (7)	7 (7)
	Ethnicity					
		Māori	8 (32)	11 (18)	4 (27)	23 (23)
		Pacific	17 (68)	50 (82)	11 (73)	78 (77)
Total participants at the 29 practices who agreed to review			45	57	110	212
	Age					
		< 60 years	4 (9)	1 (2)	-	5 (2)
		60–69 years	36 (80)	37 (65)	79 (72)	152 (72)
		70–79 years	5 (11)	19 (33)	31 (28)	55 (26)
	Gender					
		Male	27 (60)	42 (74)	68 (62)	137 (65)
		Female	18 (40)	15 (26)	42 (38)	73 (35)
	Ethnicity					
		Māori	37 (82)	45 (79)	110 (100)	192 (91)
		Pacific	8 (18)	12 (21)	0 (0)	20 (9)
	Enrolment status with PCP					
		Enrolled with same PCP	31 (69)	43 (75)	19 (17)	93 (44)
		Casual, transferred or not enrolled with that PCP	11 (24)	10 (18)	66 (60)	87 (41)
		Deceased	3 (7)	3 (5)	21 (19)	27 (13)
		Not showing up in search	0 (0)	1 (2)	4 (4)	5 (2)
Total participants currently enrolled			31	43	19	93
	Stage 2 PCP smoking status					
		Current smoker	5 (16)	1 (2)	4 (21)	10 (11)
		Ex-smoker	23 (74)	13 (30)	6 (32)	42 (45)
		Never smoker	2 (6)	29 (67)	8 (42)	39 (42)
		Missing	1 (3)	0 (0)	1 (5)	2 (2)
	Concordance (remained unchanged) of Stage 1 and Stage 2 PCP smoking data (%)		74	67	5	57
	Concordance of AAA smoking data and Stage 2 PCP smoking data (%)		16	33	95	40

The age and sex distribution among those with missing PCP smoking data did not differ significantly from those with non-missing data, but there was a significantly higher probability of missing smoking data for Māori than Pacific (*p* < 0.0001). Also, among non-deceased with enrolment status data, the proportion with missing smoking data was significantly lower in those enrolled with the same practice when reviewed (20% vs. 76%) compared with casual or transferred patients (Table 3).

Of the 212 participants from the participating PCPs, nearly half (93 participants, 44%) remained currently enrolled with that PCP. Of the 56% no longer enrolled in that PCP, 41% may have changed PCP, 13% were deceased, and 2% did not appear in the PCP data search (Table 3).

Smoking status had been changed since the initial extract in Stage 1 for 40 of the 93 participants (43%) still enrolled (Table 3). Smoking status of two of the 93 participants was missing in the Stage 2 PCP data. With these changes, the Stage 2 PCP smoking data had become concordant with AAA data in 37 participants, in addition to the 1415 participants who were concordant in the Stage 1 PCP data (Supplementary Table 2).

**Fig. 1:**
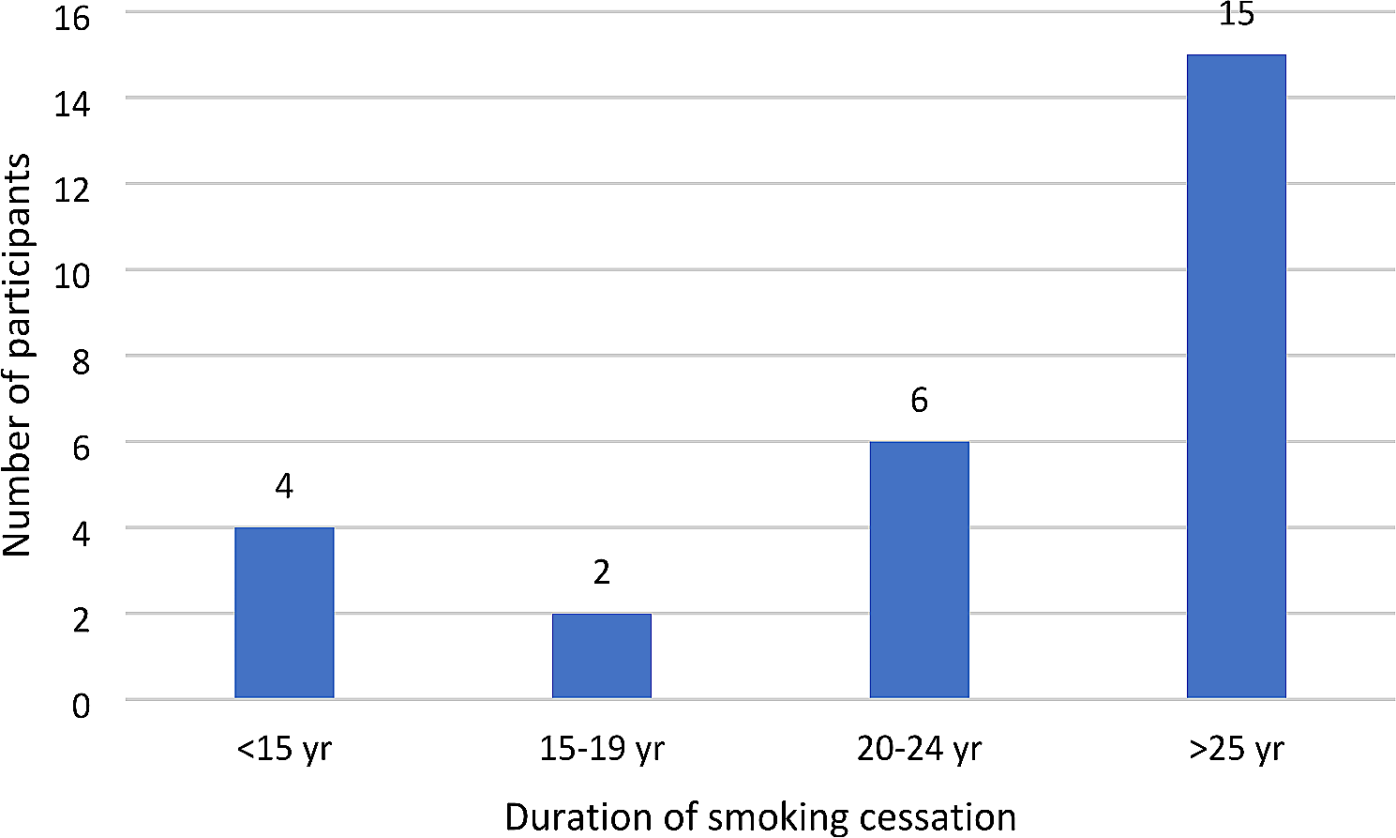
AAA data on duration of smoking cessation among ex-smokers who were classified as never smoker in Stage 2 PCP smoking data (*n* = 27)

Of the people who were ex-smokers at the time of AAA screening and were classified as never smoker in the Stage 2 PCP data (*n* = 27; Supplementary Table 2), the average duration of smoking cessation was 29 years (range 0.04-50 years) (Fig. 1). Over half (56%) had quit smoking over 25 years ago; however, 15% had quit within the last 15 years (Fig. 1). One person (4%) had quit within the last 5 years, and two people (7%) had quit within the last 10 years.

Among smokers (*n* = 10; Supplementary Table 2), 50% had smoking quantity documented but few data were recorded on years of smoking (Table 4). For ex-smokers (*n* = 42; Supplementary Table 2), over half (57%) had quit date documented but few data were recorded on quantity or years of smoking (Table 4).

**Table 4:** Completeness of recording of smoking information for those enrolled with the primary care practice

**Smokers, total**		**10**
	Quantity documented	5 (50%)
	Years smoking documented	2 (20%)
	Quantity + years smoking documented	1 (10%)
**Ex-smokers, total**		**42**
	Quantity documented	7 (17%)
	Years smoking documented	5 (12%)
	Quit date documented	24 (57%)
	Quantity, years smoking and quit date documented	4 (10%)

**Fig. 2:**
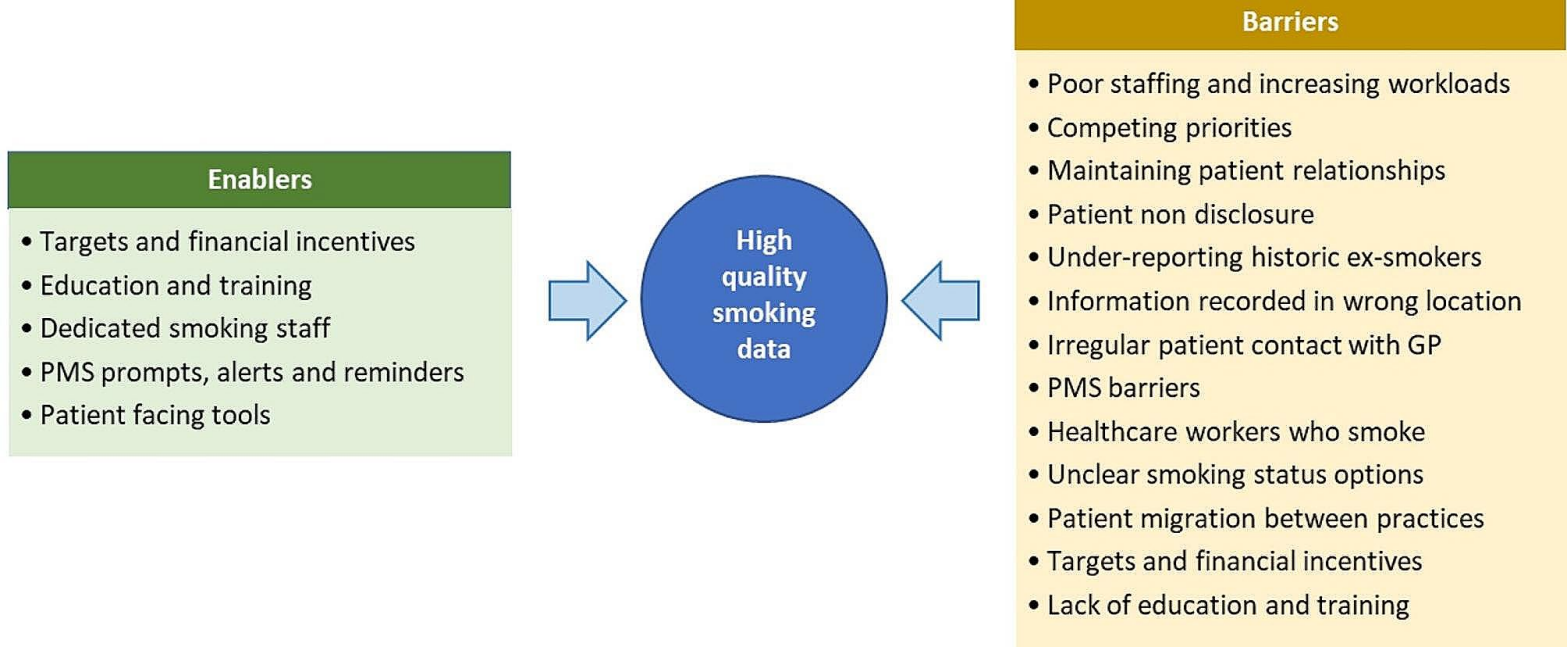
Enablers and barriers for collecting high quality smoking data in PCP

Smoking information was found recorded in various locations in the PMS including the enrolment form, social history, notes, diagnosis, screening, and external dashboards. As a result of field observations and informal conversations with PCP staff, a number of enablers and barriers to collecting high quality smoking data were identified (Fig. 2), including patient aspects such as non-disclosure and under-reporting; staff aspects such as heavy workload and staff being a smoker; and system aspects such as targets and incentives.

## Discussion

We reviewed the smoking data for a population eligible for the AAA screening research, including 1716 eligible people, predominantly contributed by participants who were 60–69 years old, male and Māori. We analysed the accuracy of smoking data recorded at primary care practices (PCPs) compared to the AAA screening research programme data before and after the screening session. On the Stage 1 PCP data extract, the smoking status in PCP and AAA screening data showed substantial agreement (82%; weighted kappa 0.76) [36] although AAA screening data systematically identified more smokers and ex-smokers than PCP data. The relative accuracy of PCP smoking data is comparable to international studies: a US study showed 85% concordance between tumour registry smoking records and electronic health records [37], an Australian study observed 82% agreement between medical records and self-reported smoking data [38], and a UK study reported 75.3% agreement between self-reported smoking status with primary care records [39].

On review of longitudinal PCP smoking records after the AAA screening session for 93 participants who were still enrolled in the PCP, smoking status of 43% of participants had been changed. The improvement in the accuracy of smoking data over the period of the audit was largely driven by missing data having been updated – an improvement in the completeness of the smoking data. However, 56 (60%) of 93 participants remained discordant with the AAA data; if the AAA data is assumed to be accurate then patients misclassified as never smokers were at risk of missing out on potentially life-saving AAA screening. Having fewer missing smoking status in the Stage 2 PCP data and the improved concordance with smoking status at AAA screening show that recording of PCP smoking data had improved over time. It is likely that the Ministry of Health and primary care performance target focus on up-to-date recording of smoking status for offer of cessation reduced the missing data over the time period between the AAA screening and the Stage 2 audit.

Detailed smoking information, such as quantity and duration of smoking, as well as quit date for ex-smokers, was largely incomplete in the PCP records. Complete and accurate detailed information is important in identifying individuals’ disease risk, particularly for programmes such as lung cancer screening – however collection of these additional data has not been a focus of primary care smoking guidelines or initiatives. Research has established that the associations of smoking with lung cancer, CVD, COPD and AAA are dose-dependent rather than binary [10, 40–43]. For example, a smoker of 35 years has 8 times the risk of AAA than a smoker of 10 years duration [41]. Risk assessment tools, such as the PLCOm2012 model for lung cancer, require information on duration of smoking, number of cigarettes smoked per day and time since cessation [8]. Only 10% of smokers and ex-smokers had complete smoking information documented in their PCP records.

The date of smoking cessation is an important factor for determining the disease risk of an ex-smoker as risk does not return to baseline immediately after quitting. The risk of developing a AAA returns to that of a never smoker after 25 years of smoking cessation [10], while the risk of developing lung cancer remains twice as high as that of a never smoker after 25 years [44] and returns to baseline after 40 years of smoking cessation [45]. Similarly, the risk of cardiovascular mortality remains after 20 years of smoking cessation [40]. In our review, among the 27 ex-smokers in AAA screening data who were misclassified as never smoker in the Stage 2 PCP data, almost half had quit smoking less than 25 years ago and four had quit less than 15 years ago. The latter group would miss the opportunity of being screened for lung cancer (if they were otherwise eligible) as they would not be considered a smoker [8, 46]. It is likely to misclassify ex-smokers as non-smokers or have missing smoking status when the person quit smoking at an early age or a long time ago [47]. International studies have predicted that 30–54% of eligible individuals could miss lung cancer screening if electronic health records alone were used to determine their eligibility [48, 49]. Identifying those with misclassified never-smoker or missing smoking status is crucial for invitation to lung cancer screening with equitable benefit [39].

Smoking status can be dynamic and recording accurate and complete smoking data in PMS may be challenging for a variety of reasons [50]. The PCP staff identified several barriers to collecting quality smoking information. These included staff shortage, increased workloads, competing priorities on other health targets, lack of standardised locations for recording or displaying data in the PMS or patient dashboard, variation in the ability or expectation of recording detailed smoking information in different PCPs, intermittent patient visits to the PCP, patient movement between PCPs, and lack of staff training on how to record smoking information. Additionally, the PCP staff reported patient discomfort when asked about their smoking status, and commented on perceptions of stigma, leading some staff members to avoid asking about smoking history to maintain patient trust. Similar barriers have been reported in other studies [51, 52]. The smoking cessation literature comments on ‘patient non-disclosure’, when smoking is regarded as socially-undesirable, which results in self-reported smoking prevalence underestimated by up to 47% [53–57]. Staff members who smoke are less likely to ask smoking history, and smoking prevalence among healthcare staff ranges from 2 to 18% [58, 59]. Another barrier to accuracy was the use of “non-smoker”, instead of “never smoker”, as non-smoker may be confused with ex-smoker.

Mitigating these barriers may enable quality improvement in recording of smoking data. Additionally, PCP staff members found prompts, alerts, and reminders useful to complete smoking information, as reported in other studies [60]. Making the collection of smoking information mandatory, with clear designated fields for data entry, may also enhance recording. Studies have shown that patients enjoy reviewing and updating their own health information on patient facing online systems [61–63]. These could allow patients to update their own smoking status, although such data would need to be interpreted with caution given the known under-ascertainment of smokers. A US study found that 54.5% of patients had implausible changes in their smoking status, such as current smoker to never smoker [64]. A recent large UK study found that individuals with missing smoking data in PCP were more likely to have a history of smoking [65]. Programmes that include PCP based eligibility criteria should consider ways to inform those recorded as non-smokers or those with missing records about the programme, and invite them to contact the programme via low cost mechanisms such as text messaging, electronic portal notification, or letters; notifying potentially eligible patients of the programme and smoking eligibility criteria, and that if they believe their data is incorrectly recorded or that they may be eligible (e.g. for lung cancer screening or AAA screening) [65, 66]. Studies have observed the unreliability of self-reported smoking status and looked at ways to tackle the underestimation of true smoking using objective measurements of smoking exposure, such as measurements of blood or urinary biomarkers [23, 67–71].

This project had some limitations. We used AAA smoking records as the standard dataset for comparison; however, it is possible that AAA smoking status may also be misclassified; it is possible that participants may have modified their smoking status if they believed it was relevant to further assessments following screening. In the Stage 1 PCP smoking data, the timing of the PCP data extract and the AAA session was variable due to site-specific extraction issues and scheduling of the AAA screening session. At Stage 2, we could not include one-third of participants from the eight non-participating PCPs, which had a high proportion of Pacific Peoples. The audit was conducted in the COVID-19 pandemic, where Pacific PCPs were heavily involved in COVID-19 community vaccination efforts and this likely contributed to their non-participation in the audit. Only one team member reviewed the longitudinal smoking records, which may result in missing some smoking records despite best efforts. Given the sample was relatively small and limited to Māori and Pacific Peoples, and recording of smoking data may vary substantially between practices [39], the results may have limited application to the general population. We reported enablers and barriers to collecting quality smoking information based on informal discussions with PCP staff rather than the systematically collected data.

## Conclusion

The smoking data at PCPs had 82% concordance (adjusted kappa 0.76) with that recorded in the Māori and Pacific AAA screening research programme, however PCP data identified fewer current and ex-smokers, and had more missing data. The audit of longitudinal smoking status, including current recorded status, for the subgroups of discordant (potentially misclassified current and ex-smokers) and missing data demonstrated improved concordance in Stage 2, suggested PCP smoking data quality had improved over time. Misclassified patients may miss invitation to smoking risk-related programmes that are based on current/ex-smoker eligibility. The New Zealand Ministry of Health should consider specific advice on the need for further PCP data quality improvement to ensure eligible individuals have their smoking-related disease risk accurately identified, and are able to benefit from screening and intervention programmes. Programmes based on smoking status should also consider additional mechanisms to notify potential participants given the level of misclassification identified in this project and in comparable health systems.

### Electronic supplementary material

Below is the link to the electronic supplementary material.

Supplementary Material 1

## Data Availability

The data used and analysed during the current study contain identifiable individual patient information. The data are not publicly available due to the data confidentiality and privacy restrictions but are available from the corresponding author on reasonable request and corresponding approvals.

## Abbreviations

AAA: Abdominal Aortic Aneurysm
AF: Atrial Fibrillation
COPD: Chronic Obstructive Pulmonary Disease
CVD: Cardiovascular Disease
GP: General Practitioner
PCP: Primary Care Practice
PMS: Patient Management System

## References

[1] US Department of Health and Human Services. The Health Consequences of Smoking — 50 Years of Progress: A Report of the Surgeon General. Centers for Disease Control and Prevention (US). 2014. https://doi.org/NBK179276. 24455788

[2] Ministry of Health. Longer, Healthier Lives: New Zealand’s Health 1990–2017. Wellington: 2020.

[3] Institute for Health Metrics and Evaluation. VizHub - GBD Compare 2019. https://vizhub.healthdata.org/gbd-compare/ (accessed March 12, 2024).

[4] Ministry of Health. Annual Update of Key Results 2022/23. New Zealand Health Survey 2023. https://minhealthnz.shinyapps.io/nz-health-survey-2022-23-annual-data-explorer/ (accessed March 12, 2024).

[5] Ministry of Health. Mortality web tool. New Zealand Mortality Collection 2021. https://minhealthnz.shinyapps.io/mortality-web-tool/ (accessed March 15, 2022).

[6] Pylypchuk R, Wells S, Kerr A, Poppe K, Riddell T, Harwood M (2018). Cardiovascular disease risk prediction equations in 400 000 primary care patients in New Zealand: a derivation and validation study. Lancet.

[7] Ministry of Health. Cardiovascular Disease Risk Assessment and Management for Primary Care 2018.

[8] Tammemägi MC, Katki HA, Hocking WG, Church TR, Caporaso N, Kvale PA (2013). Selection criteria for lung-Cancer screening. N Engl J Med.

[9] Parker K, Colhoun S, Bartholomew K, Sandiford P, Lewis C, Milne D (2023). Invitation methods for Indigenous New Zealand Māori in lung cancer screening: protocol for a pragmatic cluster randomized controlled trial. PLoS ONE.

[10] Aune D, Schlesinger S, Norat T, Riboli E (2018). Tobacco smoking and the risk of abdominal aortic aneurysm: a systematic review and meta-analysis of prospective studies. Sci Rep.

[11] Sandiford P, Grey C, Salvetto M, Hill A, Malloy T, Cranefield D (2020). The population prevalence of undetected abdominal aortic aneurysm in New Zealand Māori. J Vasc Surg.

[12] Ministry of Health. The Integrated performance and incentive Framework (IPIF): a healthy start. Best Pract J 2015.

[13] Ministry of Health. Enrollment with a general practice and primary health organisation 2022. https://www.health.govt.nz/our-work/primary-health-care/about-primary-health-organisations/enrollment-general-practice-and-primary-health-organisation.

[14] Ministry of Health. Indicator: Visited or talked to GP in past 12 months. New Zealand Health Survey 2021-22 2023. https://minhealthnz.shinyapps.io/nz-health-survey-2021-22-annual-data-explorer/_w_1eb7d253/#!/explore-indicators (accessed June 15, 2023).

[15] Best Practice Advocacy Centre New Zealand. Smoking status and cessation support. BPJ 2011.

[16] Selak V, Wells S, Whittaker R, Stewart A (2006). Smoking status recording in GP electronic records: the unrealised potential. J Innov Health Inf.

[17] Sinclair G, Kerr A (2006). The bold Promise Project: a system change in primary care to support cardiovascular risk screening. N Z Med J.

[18] Sheerin I, Hamilton G, Humphrey A, Scragg A (2007). Issues in the assessment of cardiovascular risk in selected general practices in Canterbury, New Zealand. N Z Med J.

[19] The Best Practice Advocacy Centre New Zealand. Smoking prevention and cessation in adolescents: changing futures, saving lives. Best Pract 2013:32–9.

[20] Ministry of Health. Better Help for Smokers to Quit (Primary Care) 2021/22 Q4 - Final Results by DHBs (Unpublished) 2022.

[21] Garies S, Cummings M, Quan H, McBrien K, Drummond N, Manca D (2020). Methods to improve the quality of smoking records in a primary care EMR database: exploring multiple imputation and pattern-matching algorithms. BMC Med Inf Decis Mak.

[22] Scheuermann TS, Richter KP, Rigotti NA, Cummins SE, Harrington KF, Sherman SE (2017). Accuracy of self-reported smoking abstinence in clinical trials of hospital-initiated smoking interventions. Addiction (Abingdon England).

[23] Shipton D, Tappin DM, Vadiveloo T, Crossley JA, Aitken DA, Chalmers J (2009). Reliability of self reported smoking status by pregnant women for estimating smoking prevalence: a retrospective, cross sectional study. BMJ.

[24] Djordjevic MV, Stellman SD, Zang E (2000). Doses of nicotine and lung carcinogens delivered to cigarette smokers. J Natl Cancer Inst.

[25] Von Elm E, Altman DG, Egger M, Pocock SJ, Gøtzsche PC, Vandenbroucke JP (2007). The strengthening the reporting of Observational studies in Epidemiology (STROBE) statement: guidelines for reporting observational studies. Epidemiology.

[26] Chiang N, Jain JK, Hulme KR, Vasudevan T (2018). Epidemiology and outcomes of abdominal aortic aneurysms in New Zealand: a 15-Year experience at a Regional Hospital. Ann Vasc Surg.

[27] Nair N, Shaw C, Sarfati D, Stanley J. Abdominal aortic aneurysm disease in New Zealand: Epidemiology and burden between 2002 and 2006. N Z Med J 2012;125. 22382252

[28] Sandiford P, Mosquera D, Bramley D (2012). Ethnic inequalities in incidence, survival and mortality from abdominal aortic aneurysm in New Zealand. J Epidemiol Community Health.

[29] Rossaak JI, Sporle A, Birks CL, Van Rij AM (2003). Abdominal aortic aneurysms in the New Zealand Maori population. Br J Surg.

[30] Ministry of Health. Recording smoking status 2023. https://www.health.govt.nz/our-work/preventative-health-wellness/smokefree-2025/information-practitioners-patients-who-are-quitting-smoking/recording-smoking-status (accessed March 12, 2024).

[31] Ministry of Health. Indicator Interpretation Guide 2015/16 New Zealand Health Survey. Wellington: 2016.

[32] Health New Zealand. Identity standards 2023. https://www.tewhatuora.govt.nz/our-health-system/digital-health/data-and-digital-standards/approved-standards/identity-standards/ (accessed March 12, 2024).

[33] Health New Zealand. Mortality Collection. Ministry of Health 2023. https://www.tewhatuora.govt.nz/our-health-system/data-and-statistics/nz-health-statistics/national-collections-and-surveys/collections/mortality-collection/ (accessed March 13, 2024).

[34] Vears DF, Gillam L, Vears D. Inductive content analysis: A guide for beginning qualitative researchers 2022;23:2022. 10.3316/INFORMIT.455663644555599.

[35] Cohen J (1968). Weighted kappa: nominal scale agreement provision for scaled disagreement or partial credit. Psychol Bull.

[36] Landis JR, Koch GG (1977). The measurement of Observer Agreement for. Categorical Data.

[37] LeLaurin JH, Gurka MJ, Chi X, Lee J-H, Hall J, Warren GW, et al. Concordance between Electronic Health Record and Tumor Registry Documentation of Smoking Status among patients with Cancer. JCO Clin Cancer Inf. 2021;518–26. 10.1200/CCI.20.00187. 10.1200/CCI.20.00187 33974447

[38] Noble N, Bryant J, Maher L, Jackman D, Bonevski B, Shakeshaft A (2021). Patient self-report versus medical records for smoking status and alcohol consumption at Aboriginal Community Controlled Health Services. Aust N Z J Public Health.

[39] Dickson JL, Hall H, Horst C, Tisi S, Verghese P, Worboys S (2022). Utilisation of primary care electronic patient records for identification and targeted invitation of individuals to a lung cancer screening programme. Lung Cancer.

[40] Mons U, Muezzinler A, Gellert C, Schottker B, Abnet CC, Bobak M (2015). Impact of smoking and smoking cessation on cardiovascular events and mortality among older adults: meta-analysis of individual participant data from prospective cohort studies of the CHANCES consortium. BMJ.

[41] Kent KC, Zwolak RM, Egorova NN, Riles TS, Manganaro A, Moskowitz AJ (2010). Analysis of risk factors for abdominal aortic aneurysm in a cohort of more than 3 million individuals. J Vasc Surg.

[42] Remen T, Pintos J, Abrahamowicz M, Siemiatycki J (2018). Risk of lung cancer in relation to various metrics of smoking history: a case-control study in Montreal. BMC Cancer.

[43] Lee PN, Forey BA, Coombs KJ (2012). Systematic review with meta-analysis of the epidemiological evidence in the 1900s relating smoking to lung cancer. BMC Cancer.

[44] Tindle HA, Duncan MS, Greevy RA, Vasan RS, Kundu S, Massion PP et al. Lifetime Smoking History and Risk of Lung Cancer: Results From the Framingham Heart Study. JNCI: Journal of the National Cancer Institute. 2018. 10.1093/jnci/djy041. 10.1093/jnci/djy041 PMC6235683 29788259

[45] Reitsma M, Kendrick P, Anderson J, Arian N, Feldman R, Gakidou E (2020). Reexamining rates of decline in Lung Cancer Risk after Smoking Cessation. A Meta-analysis. Ann Am Thorac Soc.

[46] Tammemägi MC, Ruparel M, Tremblay A, Myers R, Mayo J, Yee J (2022). USPSTF2013 versus PLCOm2012 lung cancer screening eligibility criteria (International Lung Screening Trial): interim analysis of a prospective cohort study. Lancet Oncol.

[47] Marston L, Carpenter JR, Walters KR, Morris RW, Nazareth I, White IR (2014). Smoker, ex-smoker or non-smoker? The validity of routinely recorded smoking status in UK primary care: a cross-sectional study. BMJ Open.

[48] Cole AM, Pflugeisen B, Schwartz MR, Miller SC (2018). Cross sectional study to assess the accuracy of electronic health record data to identify patients in need of lung cancer screening. BMC Res Notes.

[49] Modin HE, Fathi JT, Gilbert CR, Wilshire CL, Wilson AK, Aye RW (2017). Pack-year cigarette smoking history for determination of Lung Cancer Screening eligibility. Comparison of the Electronic Medical Record versus a Shared decision-making conversation. Ann Am Thorac Soc.

[50] Brown L, Agrawal U, Sullivan F. Using Electronic Medical Records to identify potentially eligible study subjects for Lung Cancer screening with biomarkers. Cancers (Basel). 2021;13. 10.3390/CANCERS13215449. 10.3390/cancers13215449 PMC8582572 34771612

[51] Sharpe T, Alsahlanee A, Ward KD, Doyle F (2018). Systematic review of clinician-reported barriers to Provision of Smoking Cessation interventions in Hospital Inpatient settings. J Smok Cessat.

[52] Ministry of Health. The New Zealand Guidelines for Helping People to Stop Smoking Update. Wellington: 2021.

[53] Gorber SC, Schofield-Hurwitz S, Hardt J, Levasseur G, Tremblay M (2009). The accuracy of self-reported smoking: a systematic review of the relationship between self-reported and cotinine-assessed smoking status. Nicotine Tob Res.

[54] Fendrich M, Mackesy-Amiti ME, Johnson TP, Hubbell A, Wislar JS (2005). Tobacco-reporting validity in an epidemiological drug-use survey. Addict Behav.

[55] Pérez-Stable EJ, Marín BV, Marín G, Brody DJ, Benowitz NL (1990). Apparent underreporting of cigarette consumption among Mexican American smokers. Am J Public Health.

[56] Ford RP, Tappin DM, Schluter PJ, Wild CJ. Smoking during pregnancy: how reliable are maternal self reports in New Zealand? J Epidemiol Community Health (1978). 1997;51(246–51). 10.1136/jech.51.3.246. 10.1136/jech.51.3.246 PMC1060468 9229052

[57] Attebring MF, Herlitz J, Berndt AK, Karlsson T, Hjalmarson A (2001). Are patients truthful about their smoking habits? A validation of self-report about smoking cessation with biochemical markers of smoking activity amongst patients with ischaemic heart disease. J Intern Med.

[58] Edwards R, Tu D, Stanley J, Martin G, Gifford H, Newcombe R (2018). Smoking prevalence among doctors and nurses-2013 New Zealand census data. N Z Med J.

[59] Duaso MJ, McDermott MS, Mujika A, Purssell E, While A (2014). Do doctors’ smoking habits influence their smoking cessation practices? A systematic review and meta-analysis. Addiction.

[60] Brinson D. How to increase the delivery of effective smoking cessation treatments in primary care settings: guidance for doctors, nurses, other health professionals and healthcare organisations 2009.

[61] Pyper C, Amery J, Watson M, Crook C, Pyper C, Amery J (2004). Patients’ experiences when accessing their on-line electronic patient records in primary care. Br J Gen Pract.

[62] Dullabh PM, Sondheimer NK, Katsh E, Evans MA (2014). How patients can improve the Accuracy of their Medical records. EGEMs.

[63] Staroselsky M, Volk LA, Tsurikova R, Pizziferri L, Lippincott M, Wald J (2006). Improving electronic health record (EHR) accuracy and increasing compliance with health maintenance clinical guidelines through patient access and input. Int J Med Inf.

[64] Polubriaginof F, Salmasian H, Albert DA, Vawdrey DK (2017). Challenges with Collecting Smoking Status in Electronic Health Records. AMIA Annu Symp Proc.

[65] Goodley P, Balata H, Alonso A, Brockelsby C, Conroy M, Cooper-Moss N (2023). Invitation strategies and participation in a community-based lung cancer screening programme located in areas of high socioeconomic deprivation. Thorax.

[66] Engela-Volker JS, Augusto AP, Datta S, Ling Y, Mcclure L (2021). P163 preparing wales for lung cancer screening – updating GP record smoking data using an automated text message system. Thorax.

[67] Wilcox RG, Hughes J, Roland J (1979). Verification of smoking history in patients after infarction using urinary nicotine and cotinine measurements. Br Med J.

[68] Kelley F, Charles S, Mcaughey J, Shepperd CJ (2013). Methodologies for the quantitative estimation of toxicant dose to cigarette smokers using physical, chemical and bioanalytical data. Inhal Toxicol.

[69] Wagenknecht LE, Burke GL, Perkins LL, Haley NJ, Friedman GD (1992). Misclassification of smoking status in the CARDIA study: a comparison of self-report with serum cotinine levels. Am J Public Health.

[70] Caraballo RS, Giovino GA, Pechacek TF (2004). Self-reported cigarette smoking vs. serum cotinine among U.S. adolescents. Nicotine Tob Res.

[71] Avidano Britton GR, Brinthaupt JA, Stehle JM, James GD (2004). Comparison of self-reported smoking and urinary cotinine levels in a rural pregnant Population. J Obstetric Gynecologic Neonatal Nurs.

