# Supplementary material for: Smoking data quality of primary care practices in comparison with smoking data from the New Zealand Māori and Pacific abdominal aortic aneurysm screening programme: an observational study
Source: BMC Public Health. 2024 Jun 5;24:1513. doi: 10.1186/s12889-024-19021-8 (PMC11154981; doi:10.1186/s12889-024-19021-8)
Supplement: Supplementary file 1 — Supplementary Material 1 [file 12889_2024_19021_MOESM1_ESM.docx]

**Supplementary table 1. Read codes extracted**

| Condition | Read Codes |
| --- | --- |
| Smoking | Never smoked = 1371 137L |
|  | Past smoker = 137K 137S 137F 1377 1378 1379 137A 137B 137N 137O E2513 |
|  | Current Smoker = 137R 137G 137P 137Q 137Z 137M 137J 137H 137C 137D 1372 1373 1374 1375 1376 E2510 E2511 E2512 E251z ZPSA10 |
|  | Not known = 137E 137I 137L |
| Hypertension | G2:         Hypertensive Disease and all lower level codes |
| Hyperlipidaemia | C32:       Disorders of the lipoid mechanism and all lower level codes |
| Diabetes | C10: Diabetes mellitus and all lower level codes |
| MI & PAD | Myocardial Infarction: G30 and all lower level codes; Gyu34 Gyu35 Gyu36  Peripheral artery disease: G73z G73z0 G73zz G732 and all lower level codes |
| Existing AAA | AAA and all lower level codes except G710 (Dissecting Aortic Aneurysm) |
| Cardia dysrhythmias | G71       Cardiac dysrhythmias:  G57 Cardiac dysrhythmias and all lower level codes |
| Other cardiovascular disease | G3 Ischaemic heart disease and all lower level codes except G33 (Angina pectoris)  G58 Heart failure and all lower level codes  G61 Intracerebral haemorrhage and all lower level codes  G63 Precerebral arterial occlusion and all lower level codes  G64  Cerebral Artery Occlusion and all lower level codes  G66 Stroke and Cerebrovascular Accident and all lower level codes  G73 Other peripheral vascular disease except G730 (Raynaud’s syndrome) + its lower level codes  G74 Arterial embolism and thrombosis and all lower level codes |
| CVD risk | CVD Risk value/code from CVD risk assessment |

**Supplementary table 2. Cross tabulation of Stage 2 PCP smoking status with Stage 1 PCP smoking status and AAA smoking status for 93 participants who were currently enrolled in the PCP**

|  |  | Stage 2 PCP smoking status | | | | Total | Concordance |
| --- | --- | --- | --- | --- | --- | --- | --- |
|  |  | Current smoker | Ex-smoker | Never smoker | Missing |  |  |
| Total | | 10 | 42 | 39 | 2 | 93 |  |
| Stage 1 PCP smoking status | | | |  |  |  | 57%* |
|  | Current smoker | - | - | - | - | - |  |
|  | Ex-smoker | 5 | 23 | 2 | 1 | 31 |  |
|  | Never smoker | 1 | 13 | 29 | 0 | 43 |  |
|  | Missing | 4 | 6 | 8 | 1 | 19 |  |
| AAA smoking status | | | | |  |  | 40%^ |
|  | Current smoker | 10 | 23 | 4 | 1 | 38 |  |
|  | Ex-smoker | 0 | 19 | 27 | 1 | 47 |  |
|  | Never smoker | 0 | 0 | 8 | 0 | 8 |  |
|  | Missing | - | - | - | - | - |  |
| * Concordance for this group means the smoking status remained unchanged; ^ Concordance of this group means additional agreement between PCP data and AAA data since the initial Stage 1 PCP data extract. | | | | | | | |

**Supplementary table 3. Strengthening the reporting of observational studies in epidemiology – the STROBE Statement**

|  | Item No | Recommendation | Page No |
| --- | --- | --- | --- |
| Title and abstract | 1 | (*a*) Indicate the study’s design with a commonly used term in the title or the abstract | 1 |
|  |  | (*b*) Provide in the abstract an informative and balanced summary of what was done and what was found | 1,2 |
| Introduction | | | |
| Background/rationale | 2 | Explain the scientific background and rationale for the investigation being reported | 3,4 |
| Objectives | 3 | State specific objectives, including any prespecified hypotheses | 4 |
| Methods | | | |
| Study design | 4 | Present key elements of study design early in the paper | 4 |
| Setting | 5 | Describe the setting, locations, and relevant dates, including periods of recruitment, exposure, follow-up, and data collection | 5,6,7 |
| Participants | 6 | (*a*) Give the eligibility criteria, and the sources and methods of selection of participants | 5,6 |
| Variables | 7 | Clearly define all outcomes, exposures, predictors, potential confounders, and effect modifiers. Give diagnostic criteria, if applicable | 6 |
| Data sources/ measurement | 8* | For each variable of interest, give sources of data and details of methods of assessment (measurement). Describe comparability of assessment methods if there is more than one group | 5,6,7 |
| Bias | 9 | Describe any efforts to address potential sources of bias | 7 |
| Study size | 10 | Explain how the study size was arrived at | 5 |
| Quantitative variables | 11 | Explain how quantitative variables were handled in the analyses. If applicable, describe which groupings were chosen and why | 6 |
| Statistical methods | 12 | (*a*) Describe all statistical methods, including those used to control for confounding | 7 |
|  |  | (*b*) Describe any methods used to examine subgroups and interactions | 7 |
|  |  | (*c*) Explain how missing data were addressed | 7 |
|  |  | (*d*) If applicable, describe analytical methods taking account of sampling strategy | 7 |
|  |  | (*e*) Describe any sensitivity analyses | 7 |
| Results | | | |
| Participants | 13* | (a) Report numbers of individuals at each stage of study—eg numbers potentially eligible, examined for eligibility, confirmed eligible, included in the study, completing follow-up, and analysed | 8 |
|  |  | (b) Give reasons for non-participation at each stage | 13 |
|  |  | (c) Consider use of a flow diagram | NA |
| Descriptive data | 14* | (a) Give characteristics of study participants (eg demographic, clinical, social) and information on exposures and potential confounders | 8 |
|  |  | (b) Indicate number of participants with missing data for each variable of interest | 8-10 |
| Outcome data | 15* | Report numbers of outcome events or summary measures | 8 |
| Main results | 16 | (*a*) Give unadjusted estimates and, if applicable, confounder-adjusted estimates and their precision (eg, 95% confidence interval). Make clear which confounders were adjusted for and why they were included | 8,9 |
|  |  | (*b*) Report category boundaries when continuous variables were categorized | 8 |
|  |  | (*c*) If relevant, consider translating estimates of relative risk into absolute risk for a meaningful time period | NA |
| Other analyses | 17 | Report other analyses done—eg analyses of subgroups and interactions, and sensitivity analyses | 8,9 |
| Discussion | | | |
| Key results | 18 | Summarise key results with reference to study objectives | 10 |
| Limitations | 19 | Discuss limitations of the study, taking into account sources of potential bias or imprecision. Discuss both direction and magnitude of any potential bias | 12,13 |
| Interpretation | 20 | Give a cautious overall interpretation of results considering objectives, limitations, multiplicity of analyses, results from similar studies, and other relevant evidence | 10-13 |
| Generalisability | 21 | Discuss the generalisability (external validity) of the study results | 13 |
| Other information | | | |
| Funding | 22 | Give the source of funding and the role of the funders for the present study and, if applicable, for the original study on which the present article is based | 15 |

*Give information separately for exposed and unexposed groups.

Reference: von Elm, E., Altman, D. G., Egger, M., Pocock, S. J., Gøtzsche, P. C., & Vandenbroucke, J. P. (2007). The Strengthening the Reporting of Observational Studies in Epidemiology (STROBE) statement: Guidelines for reporting observational studies. *Epidemiology*, *18*(6), 800–804. https://doi.org/10.1097/EDE.0b013e3181577654
